# The association between smoking and blood pressure in men: a cross-sectional study

**DOI:** 10.1186/s12889-017-4802-x

**Published:** 2017-10-10

**Authors:** Guoju Li, Hailing Wang, Ke Wang, Wenrui Wang, Fen Dong, Yonggang Qian, Haiying Gong, Chunxia Hui, Guodong Xu, Yanlong Li, Li Pan, Biao Zhang, Guangliang Shan

**Affiliations:** 1 Department of Epidemiology and Statistics, Institute of Basic Medical Sciences, Chinese Academy of Medical Sciences, School of Basic Medicine, Peking Union Medical College, Beijing, China; 2 Inner Mongolia Center for Disease Control and Prevention, Hohhot, China; 3 National Office for Maternal and Child Health Surveillance of China, Department of Obstetrics, Key Laboratory of Birth Defects and Related Diseases of Women and Children (Sichuan University), Ministry of Education, West China Second University Hospital, Sichuan University, Chengdu, China; 4 China-Japan Friendship Hospital, Beijing, China; 5 Fangshan District Center for Disease Control and Prevention, Beijing, China; 6 Qingdao Women and Children’s Hospital, Qingdao University, Qingdao, Shandong 266011 China

**Keywords:** Blood pressure, Hypertension, Smoking, Tobacco

## Abstract

**Background:**

Cigarette smoking is a known risk factor for cardiovascular disease (CVD), but the association between smoking and blood pressure is unclear. Thus, the current study examined the association between cigarette smoking and blood pressure in men.

**Methods:**

Systolic blood pressure (SBP), diastolic blood pressure (DBP), mean arterial pressure (MAP) and pulse pressure (PP) were examined using digital blood pressure measuring device, and smoking status was determined with China National Health Survey.

**Results:**

The ANCOVA showed that the adjusted DBP and MAP were lower in current smokers versus nonsmokers and the adjusted SBP was lower in current smokers versus former smokers (*P* < 0.05). Additionally, the adjusted PP tend to be decreased steadily as the pack·years increased in current smokers. In a fully adjusted logistic regression model, former smokers had increased ORs (95% CI) of 1.48 (1.01, 2.18) of hypertension and current smokers had not increased ORs (95% CI) of 0.83 (0.61, 1.12), compared with never smokers.

**Conclusions:**

The findings revealed that the adjusted blood pressure were lower in current smokers versus nonsmokers and former smokers. No significant dose-dependent effect of current smoking on blood pressure indices except PP was observed. Smoking cessation was significantly associated with an increased risk of hypertension. However, current smoking was not a risk factor of hypertension.

## Background

Hypertension, also known as high blood pressure, was defined as systolic blood pressure (SBP) of at least 140 mmHg, or diastolic blood pressure (DBP) of at least 90 mmHg, or self-reported diagnosis of hypertension. Hypertension is a major public health problem around the world. A cross-sectional study conducted in 17 countries and 142,042 adults showed that the prevalence of hypertension was 40.8% [1], and in China the prevalence of hypertension was 41.9% [2]. Hypertension was the leading risk factor for global disease burden and accounting for 9.4 million deaths in 2010 [3]. It contributes to the burden of stroke, heart disease and kidney failure. It is responsible for at least 51% of deaths due to stroke and 45% of deaths due to heart disease [4].

In 2015, the age-standardised prevalence of daily smoking was 25.5% for men and 5.4% for women worldwide, and in China the prevalence of daily smoking was 37.5% for men and 2.2% for women [5]. The WHO estimates that about 49.3% of men and about 2.0% of women aged 15 and over smoked in China [6]. Cigarette smoking is a considerable risk to public health, being responsible for nearly 6.3 million deaths and 6.3% of global DALYs worldwide [3]. It is a major risk factor for several diseases, including lung cancer [7], coronary heart disease [8], and stroke [9]. However, the relationship between smoking and blood pressure are not univocal, with some studies showing a positive [10, 11], and others an inverse association [12, 13]. Cavusoglu reported that smoking could cause direct endothelial damage, leading to endothelial dysfunction and impairment of endothelium dependent coronary vasodilation [14]. In addition, studies have concluded that smoking produces statistically significant changes in forearm haemodynamics affecting both small and large arteries [15], damage to the endothelium [16], and thought to be important in the pathophysiology of hypertension. The American Heart Association (AHA) blood pressure measurement recommendations pointed out that clinic-based measurements that predict vascular disease include SBP and DBP, as well as mean arterial pressure (MAP) and pulse pressure (PP) [17]. To our knowledge, there is no study of the relationship between smoking and blood pressure indices in China. In this study, we aimed to investigate the association between smoking and blood pressure in men in Inner Mongolia.

## Methods

### Data

The data was derived from the “China National Health Survey (CNHS)” study and collected in 2014. This is a cross-sectional study and designed to examine the relationship of cigarette smoking and blood pressure in men. A sample aged 20–80 years was selected using a multistage cluster sampling method, including Bayan Nur, Xilingol League, Ulanqab and Hohhot, which has been extensively described early [18].

Recruited participants include residents who had been living in Inner Mongolia for more than 1 year. Subjects were regarded as Mongolian or Han people if they and their parents were all Mongolian or Han ethnicity. Of the 1334 men aged 20–80 years, we excluded 55 subjects who were not Mongolian or Han, 2 subjects aged less than 20 and 29 subjects with missing values on baseline characteristics. Ultimately, the study population consisted of 1248 subjects. When we study the association between smoking status and SBP, DBP, MAP and PP, 226 self-reported use of pharmacological medication for the hypertension that might affect blood pressure were excluded from the study.

All participants provided written informed consent. The study was approved by the Institutional Review Board of the Institute of Basic Medical Sciences, Chinese Academy of Medical Sciences.

### Health survey

A self-reported questionnaire was used to obtain the demographic information, tobacco consumption status and history of diseases. The use of trained interviewers, and the qualified program managers checks on responses of participants improved the validity of the self-reported data. Indices of blood pressure, including SBP, DBP, MAP and PP, were measured in all eligible participants. The measurements were measured by trained medical staffs under the supervision the qualified program managers.

Subjects were divided into never smokers, former smokers and current smokers, on the basis of the amount of cigarette smoking and smoking habits in the questionnaire. Current cigarette smokers were defined as those people who smoked at least one cigarette per day and last for at least 6 months. Former smokers were defined as those people who had stopped smoking more than 6 months prior to the study. Smoking pack-years are calculated as the average number of packs smoked per day multiplied by the total number of years smoked during lifetime. Smokers were classified according to current smoking habits into four categories: ‘light smokers (0.025–5 smoking pack·years)’, ‘medium smokers (5–14 smoking pack·years)’ ‘heavy smokers (14–26 smoking pack·years)’, and ‘extreme smokers (more than 26 smoking pack·years)’.

Drinking status was divided into three categories: never drinkers, light drinkers (consumption no more than 30 ml of ethanol per day) and heavy drinkers (consumption more than 30 ml of ethanol per day).

### Measurements

Height was measured to nearest 0.1 cm using fixed stadiometer, whereas weight was measured with slandered weighing scale (BC-420, TANITA, Japan). Body mass index (BMI) was calculated as the subject’s weight (kg) divided by the height squared (metres). Participants were classified to lean or healthy, overweight, and obesity according to the World Health Organization criteria. Blood pressure was measured from sitting position using standardized procedure using digital blood pressure measuring device (Omron HEM-907, Japan). MAP was calculated as (SBP + 2DBP)/3, while PP was calculated as SBP-DBP. Hypertension was defined as an average (calculated from 3 measurements) systolic blood pressure (SBP) ≥140 mmHg, or an average diastolic blood pressure (DBP) ≥90 mmHg, or self-reported diagnosis of hypertension.

### Statistical analysis

The characteristics of the groups according to the smoking status were based on the Chi-square test or One-way analysis of variance for differences among frequencies and means, respectively. The bonferroni method was used for multiple comparisons. ANCOVA tests were used to compare SBP, DBP, MAP and PP among the smoking groups, while adjusting for age, BMI, alcohol drinking and ethnicity. Trend analysis of SBP, DBP, MAP and PP according to current smoking habits was performed using General linear model analysis, current smoking habits was an independent and continuous variable. We also examine the associations between smoking status and hypertension, multivariate logistic regression analysis, using a enter method, was performed to examine the associations. All *P* values of less than .05 were considered to be statistically significant. All of the analyses were performed using SAS software version 9.2 (SAS Institute Inc., Cary, NC, USA).

## Results

In the present study, a total of 1248 individuals were included in the analysis, the mean age was 46.21 ± 14.00 years. When we stratified by smoking status, there were 220(17.63%) former smokers, and 673(53.93%) current smokers. As presented in Table 1, former smokers were older (Bonferroni, *P* < 0.0167), shorter(Bonferroni, *P* < 0.0167), and the mean BMI was higher (Bonferroni, *P* < 0.0167) when compared with nonsmokers and current smokers. There were significantly more heavy drinking current smokers compared with the never smoking category (Bonferroni, *P* < 0.0056).

**Table 1:** Characteristics of the study population according to smoking status

Variables	Never smokers (*n* = 355)	Former smokers (*n* = 220)	Current smokers (*n* = 673)	*P* value
Han,%	239(67.32)	154(70.00)	498(74.00)	0.07
Age, years	45.00 ± 14.05	55.62 ± 12.56	43.77 ± 13.14	<0.001
Height, cm	170.33 ± 6.30	169.01 ± 6.17	170.54 ± 5.93	0.005
Weight, kg	73.74 ± 12.65	75.21 ± 11.87	73.00 ± 12.57	0.07
BMI, kg/m^2^	25.39 ± 3.96	26.29 ± 3.55	25.07 ± 3.91	<0.001
BMI,%				
Lean or healthy	174(49.01)	76(34.55)	330(49.03)	0.003
Overweight	139(39.15)	115(52.27)	268(39.82)	
Obesity	42(11.83)	29(13.18)	75(11.14)	
Alcohol Drinking, %				
Never	98(27.61)	43(19.55)	109(16.20)	<0.001
Light	173(48.73)	107(48.64)	328(48.74)	
Heavy	84(23.66)	70(31.82)	236(35.07)	

Table 2 shows SBP, DBP, MAP and PP in nonsmokers, former smokers and current smokers. After adjustment for age, BMI, alcohol drinking and ethnicity, ANCOVA comparisons showed no significant differences in blood pressure indices (all *P* > 0.05), except DBP and MAP(*P* < 0.05), in current smokers versus nonsmokers. In addition, when compared with former smokers, current smokers tended to have a lower SBP (*P* < 0.05).

**Table 2:** Blood pressure indices of the study population according to smoking status

Variables	Never smokers (*n* = 303)	Former smokers (*n* = 145)	Current smokers (*n* = 574)
Systolic blood pressure, mmHg	125.81 ± 13.13	126.52 ± 13.30	124.11 ± 14.41^b^
Diastolic blood pressure, mmHg	79.65 ± 9.77	79.58 ± 9.90	78.35 ± 10.73^a^
Pulse pressure, mmHg	46.15 ± 8.90	46.94 ± 9.02	45.77 ± 9.77
Mean arterial pressure, mmHg	95.04 ± 10.19	95.22 ± 10.33	93.60 ± 11.19^a^

Data are in mean ± SD. ANCOVA tests were used to compare SBP, DBP, MAP and PP among the smoking groups, while adjusting for age, BMI, alcohol drinking and ethnicity

^a^: denotes statistical significance between Never smokers and current smokers

^b^: denotes statistical significance between former smokers and current smokers

**Fig. 1:**
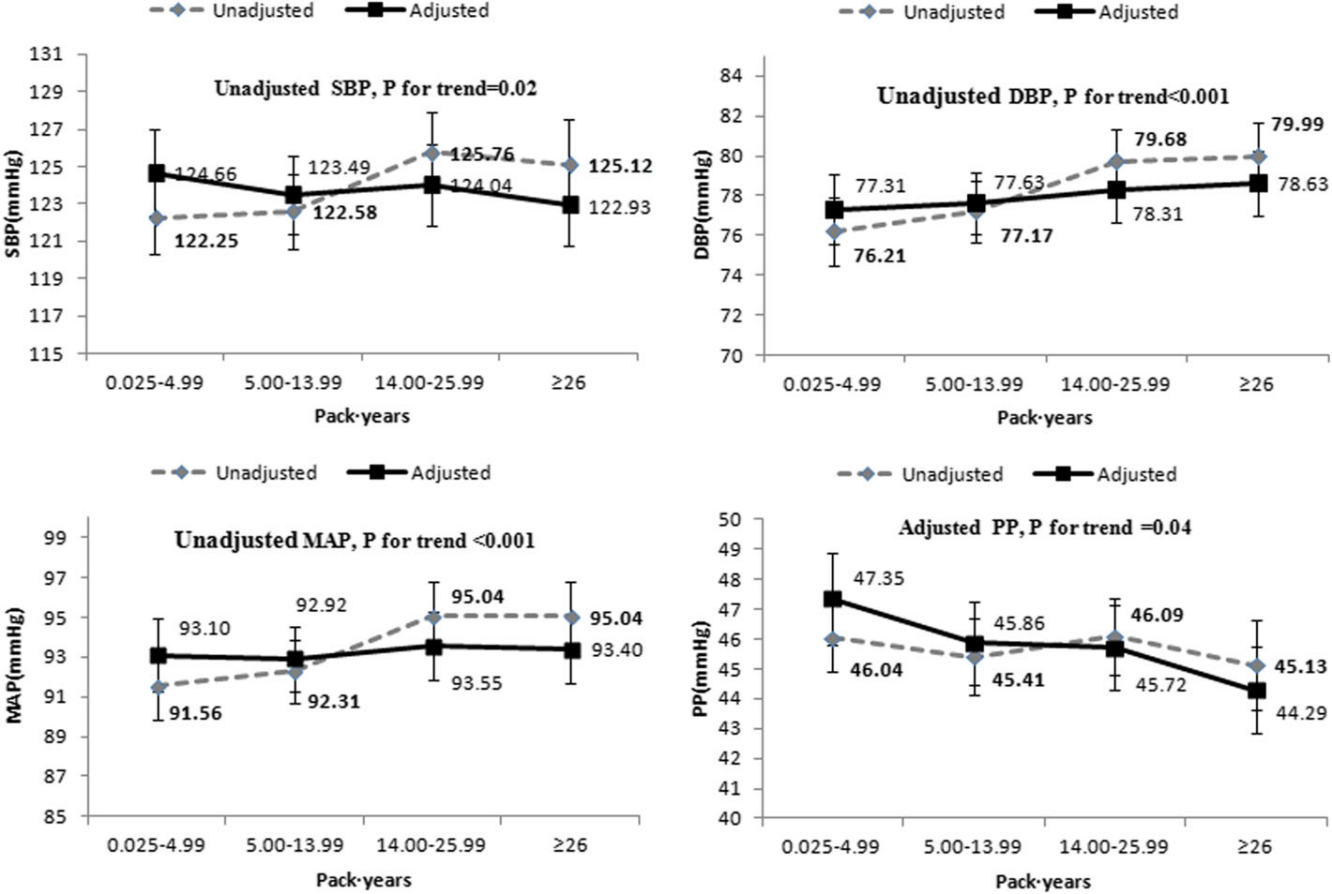
Comparison of the unadjusted and adjusted mean SBP, DBP, MAP, and PP by quartiles of pack·years of current smokers. The subjects who were taking anti-hypertensive therapy were excluded. Age, BMI, alcohol drinking and ethnicity were adjusted. Data are presented as means with 95% confidence intervals (error bars)

Fig. 1. shows the unadjusted and adjusted means of blood pressure in current smokers. The unajusted SBP, DBP and MAP increased steadily as the pack·years increased in current smokers(*P*_trend_ < 0.05). The adjusted PP decreased as the pack·years increased in current smokers (*P*_trend_ < 0.05).

In Table 3, never smokers served as the control, the unadjusted OR (95% CI) showed that the prevalence of hypertension was 2.36 (95%CI:1.67–3.34) times higher in former smokers. The age-adjusted OR (95% CI) and age, BMI-adjusted OR (95% CI) both showed that the prevalence of hypertension was higher in former smokers (OR = 1.77, 95%CI:1.23–2.54; OR = 1.55 95%CI:1.06–2.26, respectively). The multivariate models that considered age, BMI, alcohol drinking and ethnicity also showed similarly OR (95% CI) of prevalence of hypertension for former smokers (OR = 1.48, 95%CI:1.01–2.18).

**Table 3:** Association between smoking status and hypertension by multivariate logistic regression models

Variables	Never smokers (*n* = 355)	Former smokers (*n* = 220)	Current smokers (*n* = 673)
Total number of people with hypertension, %	121(34.08)	121(55.00)	206(30.61)
Unadjusted OR(95%CI)	1.00(ref)	2.36(1.67–3.34)	0.85(0.65–1.12)
Age-adjusted OR(95%CI)	1.00(ref)	1.77(1.23–2.54)	0.86(0.65–1.14)
Age, BMI-adjusted OR(95%CI)	1.00(ref)	1.55(1.06–2.26)	0.88(0.66–1.19)
Multivariate-adjusted^a^ OR(95%CI)	1.00(ref)	1.48(1.01–2.18)	0.83(0.61–1.12)

^a^ Adjusted for age, BMI, alcohol drinking and ethnicity

## Discussion

This study examined the relationship between smoking and blood pressure in men. The findings revealed that the adjusted DBP and MAP were lower in current smokers versus nonsmokers. We also found that the adjusted SBP was lower in current smokers versus former smokers. Additionally, the unadjusted SBP, DBP and MAP increased steadily as the pack·years increased in current smokers, but only the adjusted PP tend to be decreased. The multivariate models indicated that former smoking was significantly associated with an increased risk of hypertension.

Alomari reported that smoking immediately increases DBP and MAP [19]. Studies indicated that elevated nicotine mediated an increase of sympathetic nervous system activities and released of epinephrine, norepinephrine and vasopressin hormones [20–22]. However, the long term effect is controversial. A cohort study conducted in Japanese men found that the adjusted mean of change in blood pressure of current smokers was lower than in nonsmokers [23]. Another cohort study conducted in Sweden women found that the adjusted mean of change in blood pressure of current smokers was higher than in nonsmokers [24].

Many early epidemiological studies have reported that there were no significant dose-effect relationships between smoking amount and SBP or DBP [25, 26]. A study in Japanese men showed that there were no relationships between smoking amount and SBP or DBP when lifestyle and other confounding factors were considered [25]. A meta-analysis of 23 population-based studies including a total of 141,317 individuals also found that there was no causal association between smoking heaviness in current smokers and SBP or DBP [26]. A recent study has shown that two SNPs located on chromosome 14 (rs11158609) and 17 (rs8078051) significantly associated with SBP including the genetic interaction with cigarette smoking [27]. A further study between the blood pressure and genetic susceptibility is urgently needed to clarify the observation.

We have not found any epidemiological study reporting the associations between smoking amount and PP or MAP in adults. The Irbid-TRY study conducted in male adolescent showed that smoking cigarettes predicted lower SBP, DBP, MAP and PP, even when confounding factors were added to the regression model [28]. Our study also found that the adjusted SBP was lower in current smokers versus former smokers. We also found that former smoking was a risk factor in prevalence of hypertension, but the current smoking was not associated with the prevalence of hypertension in all models. Some studies have conducted that acute blood pressure increase from tobacco smoking [29] and that blood pressure decreases 1-week after smoking cessation [30]. However, there is no consensus regarding the role of smoking in long-term blood pressure in generally healthy people and the effect of smoking cessation on blood pressure remains unclear with contradictory findings [10, 23, 31–33]. A recent meta-analysis including 62 randomized controlled trials concluded that smoking cessation was associated with a mean weight gain of 4-5Kg after 1 year of abstinence [34]. Our data indicated that former smokers had a slightly higher mean BMI than current smokers and nonsmokers (*P* < 0.0167 for bonferroni test). After a careful reanalysis the data, the adjusted ORs (95% CI) of prevalence of hypertension in former smokers was 2.17 (1.52, 3.10) and this result was in accordance with our findings. We also tried to analyse the relationship between the date of hypertension diagnosed and smoking prohibiting time (*n* = 80), we found that 55% (44/80) of subjects suffer from hypertension after stop smoking. In addition, we found the prevalence of stroke and heart disease was higher in the former smokers than never smokers and current smokers (17.73% vs. 7.61 and 7.88, respectively).This may due to the high prevalence of hypertension in the former smokers. Smoking is a known risk factor of lung cancer, coronary heart disease and stroke, when subjects got this disease, they may stop smoking under the advice of doctors.

Strength of this study is that its sample is representative of the general population aged 20–80 of men. In addition, this is the first study which demonstrated the relationship between smoking and the four blood pressure indices among men in China. On the other hand, this study has limitations. This is a cross-sectional study and the data do not allow the final conclusions about causal relations. Therefore, cohort studies are needed to further understand the chronic effect of smoking on blood pressure. Furthermore, sample size was relatively small, and further studies need to examine the association between smoking and blood pressure in larger sample. In addition, BMI, age, alcohol consumption and ethnicity might be more useful. Likewise, residual confounding due to lifestyle factors cannot be excluded.

## Conclusions

The current study revealed the relationship between smoking and blood pressure indices in China. The findings revealed that the adjusted blood pressure were lower in current smokers versus nonsmokers and former smokers. No significant dose-dependent effect of current smoking on blood pressure indices except PP was observed. Smoking cessation was significantly associated with an increased risk of hypertension.

## Abbreviations

AHA: American Heart Association
BMI: Body mass index
DBP: Diastolic blood pressure
MAP: Mean arterial pressure
PP: Pulse pressure
SBP: Systolic blood pressure
